# The relations between parents’ acceptance/rejection and undergraduate adjustment to college: the moderating role of undergraduate collectivism

**DOI:** 10.3389/fpsyg.2024.1491540

**Published:** 2024-12-18

**Authors:** Hua Niu, Jie Liu, Wenji Duan, Shuna Li

**Affiliations:** ^1^College of Marxism, Shandong University of Science and Technology, Qingdao, China; ^2^Mental Health Center, Qingdao Preschool Teachers School, Qingdao, China

**Keywords:** parents’ acceptance, parents’ rejection, undergraduate adjustment to college, collectivism, moderating role

## Abstract

**Introduction:**

The present study examined the moderating effects of undergraduate collectivism in the relations between parents’ acceptance/rejection and undergraduate adjustment to college in Chinese societies.

**Methods:**

A survey was conducted covering 5,444 Chinese undergraduates and involved the use of the Parental Acceptance and Rejection Questionnaires (PARQ-short form), the College Student Adaptability Inventory (CSAI), and the Individualism-Collectivism Scale (ICS).

**Results:**

Findings revealed that undergraduate’s collectivism moderated the relations between acceptance/rejection and undergraduate adjustment to college for fathers, but not for mothers. Compared to low collectivism undergraduates, those undergraduates high in collectivism experienced stronger positive impacts from fathers’ acceptance and more pronounced negative effects from fathers’ rejection.

**Discussion:**

Findings from this study highlight the importance of considering how the cultural value (such as collectivism) may influence the relation between parenting and child development.

## Introduction

Parents’ acceptance-rejection theory tries to predict and explain the basic reasons, outcomes and other variables of being accepted and rejected by parents. As pointed by parental acceptance-rejection theory, parents’ acceptance refers to children feel loved by the parents or other caregiver parents, such as love, caress, cuddle children, share her/his feelings and meet her/his needs, whereas parents’ rejection refers to parents delay meeting the physical and mental needs of the child and become hostile toward her/him (Rohner et al., 2005a). Parents’ acceptance or rejection affects the childhood and other periods of life. Therefore, in parents’ rejection, parents fail to exhibit affinity and love toward the child, ignore her/his interest and care, and cause both physical and psychological damage in her/him (Rohner et al., 2005b). Indeed, many studies have demonstrated that higher levels of child school adjustment are commonly related with parents’ higher levels of acceptance (Chen et al., 1997; Khaleque and Rohner, 2004; Rohner, 2010; Ali et al., 2015). Conversely, as an example, a meta-analysis of 43 studies found that rejection had consistently negative effects on the psychological and behavioral functioning of both children and adults worldwide (Khaleque and Rohner, 2002).

Despite much evidence demonstrated that the perceived parents’ acceptance and rejection have a significantly stronger relation with child school adjustment, however, not all children who experienced parents’ acceptance or rejection during childhood are consequentially present high or low levels of school adjustment (Shelton and Harold, 2007; Wang et al., 2017; Ramírez-Uclés et al., 2018). In other words, the relations between parents’ acceptance or rejection and child school adjustment may moderated by other factors. Therefore, to explore the moderating factors, especially the protection factors for the association between parents’ acceptance or rejection and child school adjustment is of great significance for formulating evidence-based intervention strategies to improve child school adjustment. Indeed, to date, extensive research has confirmed that there are many moderating factors in the association between parents’ acceptance or rejection and child school adjustment, including children’s individual factors, such as children’s sex and age (Ramírez-Uclés et al., 2018) and children’s coping strategies (Shelton and Harold, 2007), family environmental factors, such as the other parent’s acceptance (Wang et al., 2017) and parents’ power and prestige (Lee and Chyung, 2014), and social environmental factors, such as children’s peer friendship (Schwartz et al., 2000), and teacher-student relationship (Huang et al., 2016). However, another possible and important factor is that cultural factor, such as children’s collectivism, may condition the effects of parents’ acceptance or rejection on child school adjustment.

Quite a bit of previous theories and research argued that collectivism may act as a protective factor against the impact of parenting on child school adjustment. To be specific, by definition, in collectivism there is a strong emphasis on interdependent relationships with others, give priority to the goals of their in-groups, shape their behavior primarily on the basis of in-group norms, and behave in a communal way (Cialdini et al., 1999; Triandis, 2001). For example, Ohbuchi et al. (1999) showed that collectivists in conflict situations are primarily concerned with maintaining their relationship with others. Thus, collectivists prefer to adopt methods which do not destroy relationships to resolve conflicts (Triandis, 2001). Moreover, from the perspective of cultural self, Markus and Kitayama (1991) pointed that individuals in different cultures have strikingly different construal of the self, which can influence individual cognition, emotion, and motivation. In this case, individual cultural values can impact individual development via shape different construal of the self. Given the above considerations, due to collectivists are value relationship, and are more likely to be shaped with interdependent view of the self with others, children high in collectivism may be more likely to interpret their parents’ rejection as acceptable or reasonable behavior, which in turn may weaken the negative effect of parents’ rejection on child school adjustment. For example, as concrete manifestations of parents’ rejection, parents’ harsh discipline is highly accepted in Chinese societies because parental psychological aggression and corporal punishment are often accepted as expressions of love and concern in traditional Chinese societies, as the Chinese proverb goes, “Beating and scolding is the emblem of love” (Chao, 1994; Liu and Wang, 2015). In other word, collectivism can serve as a protective factor against the outcome of parents’ rejection. Indeed, some studies tend to support this perspective. For instance, using cross-cultural research design, some traditional research, such as Kim (2005) found that behavioral control is related to the positive outcome of Korean adolescents and they perceive it as parents’ warmth and acceptance, whereas European American adolescents perceive behavioral control as the negative indices of parenting, thereby reducing the impact of behavioral control on child development. That is, compared with individualists, for collectivists such as Korean adolescents, perceive parents’ behavioral control is perceived as warmth and acceptance, and related to positive outcomes rather than negative outcomes. Moreover, as typical of collectivism, familism value has repeatedly linked with children adjustment. Higher levels of familism value were associated with adolescent’s higher happiness, lower internalizing and externalizing problem behaviors (Campos et al., 2014; Cahill et al., 2021).

However, some other previous theories and research argued different perspective on the impact of collectivism on the relation between parenting and child school adjustment. Collectivism may be more likely to act as an amplifier factor rather than a protective factor, since the collectivists’ characteristics of emotional sensitivity and attributions. Specifically, collectivists are more sensitive to the emotions of others than individualists (Markus and Kitayama, 1991; Huppert et al., 2018). In this context, compared with individualists, collectivists are more highly sensitive to the parents’ emotion expression in the process of parent–child interaction, such as warmth and care in parents’ acceptance, or anger and apathy in parents’ rejection. As pointed by parental acceptance-rejection theory and existing research, parents’ acceptance is regarded as a positive factor for individual development, while parents’ rejection is regarded as a negative factor for individual development (Khaleque and Rohner, 2002; Rohner et al., 2005a; Rohner, 2010). Thus, children high in collectivism are more susceptible to parents’ rejection and also benefit more from parents’ acceptance. Alternatively, regarding to attributions, as pointed by Oyserman et al. (2002), collectivists implies that judgment, reasoning, and causal inference generally oriented toward the situation or social context rather than the person. In this context, compared with individualists, collectivists are more likely to make self-attributions about parents’ rejection and acceptance rather than parents’ disciplinary strategies, and are therefore more likely to lower self-evaluation due to rejection or increase self-evaluation due to acceptance, leading to negative or positive development outcomes. Thus, compared with individualism collectivism may lead both the advantageous effect of parents’ acceptance and detrimental effect of parents’ rejection are more significantly.

To the best of our knowledge, presently there is no study that has actually concurrently examined the moderating effect of collectivism on the relation between parents’ acceptance and child school adjustment, and on the relation between parents’ rejection and child school adjustment. Accordingly, it is necessary to investigate whether collectivism could act a protective factor or an amplifier factor on the effects of parents’ acceptance or rejection on child school adjustment.

Notably, previous research was mainly focuses on the macro perspective, attempt to investigate the difference between individualism and collectivism by conducting cross-cultural research. For example, with a large sample of children from 13 diverse countries, Huppert et al. (2018) found that children from the more individualist cultures favored equitable distributions at an earlier age than children from more collectivist cultures overall. Despite Markus and Kitayama (1991) pointed that individuals in the same cultural group may be not hold the same cultural values, few studies have actually treated cultural values as continuous variables rather than simple dichotomies, and examine the possible moderating role of continuous changes in collectivism in the relations between parents’ acceptance or rejection and child school adjustment in a same cultural group. Moreover, research which investigate the effect and potential mechanism of parents’ acceptance or rejection on child school adjustment was mainly focuses on individuals during childhood (Trentacosta and Shaw, 2009; Peters et al., 2011; Putnick et al., 2015; Ramírez-Uclés et al., 2018). By contrast, there have been few studies examining the long-term effects of early parents’ acceptance or rejection on child school adjustment in adolescents. Considering that the effect of parents’ acceptance or rejection on child school adjustment is a long-term process, there may exist a sleeper effect (Howell, 2011; Vu et al., 2016). That is, parents’ acceptance or rejection has minimal or no immediate impact on child school adjustment, but the effect gradually becomes more significant or noticeable after a period of time (Howell, 2011; Vu et al., 2016).

To address limitations in the existing literature, the present study aimed to investigate the moderating effects of undergraduate’s collectivism of the relations between both mothers’ and fathers’ parenting (acceptance and rejection) and undergraduate adjustment to college with a relatively large sample of Chinese undergraduate. The present study had three hypotheses. First, both fathers’ and mothers’ acceptance were expected to positively influenced the undergraduate adjustment to college, while both fathers’ and mothers’ rejection were expected to have a negative impact on the undergraduate adjustment to college. Second, the undergraduate’s collectivism was expected to moderate the links between both parents’ parenting (acceptance and rejection) and undergraduate adjustment to college. Compared to undergraduate low in collectivism, those high in collectivism experienced a stronger positive impact from parents’ acceptance and a more pronounced negative effect from parents’ rejection. Three, the pattern of moderating effect of collectivism may be similar in fathers’ model and in mothers’ model.

## Methods

### Participants

The present study was conducted with an original sample of 5,818 college students from an engineering undergraduate university in the city of Qingdao, in Shandong Province, Eastern China. Data from 175 (3.01%) participants were not included in the analyses due to consistent answer, or the time taken to complete the assessment too long or too short, which resulted in a sample of 5,643 college students (35.73% female, and 64.27% male) with an average age of 18.21 years (*SD* = 0.70 years; range from 15 to 24 years). Further, 15 college students under the age of 17 and 184 college students over the age of 19 were not included in the analyses, which resulted in a final sample of 5,444 college students (35.65% female, and 64.35% male) with an average age of 18.15 years (*SD* = 0.54 years; range from 19 to 19 years). Data on participants’ socioeconomic status indicated that the sample was largely composed of working and middle-class families. To be specific, 76.53% of the college students’ mothers and 72.37% of the college students’ fathers were employed at working class jobs (e.g., farmer, factory workers), and 23.47% of the mothers and 27.63% of the fathers held a professional, managerial, or technical position (e.g., teachers, doctors, and civil servants). In terms of the college students’ parents’ education level, 24.36% of college students’ mothers and 12.91% of college students’ fathers had completed elementary school education or lower, 36.50 and 40.22% were middle school graduates, 24.32 and 27.15% were high school graduates, and 14.83 and 19.31% were college graduates or higher.

### Procedure

Participants in the present study were recruited from an engineering undergraduate university in the city of Qingdao, in Shandong Province, Eastern China. When the informed consent was obtained, the data in the present study was collected with a mental health survey taking a class as a unit. Specifically, undergraduates were led to the mental health assessment center of the university as a unit of the class and sit in front of the computer. The undergraduates were told that the results of the test were neither good nor bad, and the results would be kept strictly confidential. Undergraduates were requested to complete the questionnaires on the computer independently. The psychometric system checks and indicates whether all questions have been completed and records the time taken to complete the assessment. Institutional Review Board of Shandong University of Science and Technology approved the data collection procedures.

### Measures

#### Parents’ acceptance-rejection

The short form of Parental Acceptance and Rejection Questionnaires (PARQ-short form) was used to assess parents’ acceptance and rejection in the present study (Rohner et al., 2005a,b), which consisted 24 items assessed on a 4-point Likert-type scale ranging from 1 (almost never true) to 4 (almost always true). Specifically, 8 items in original warmth/affection subscales were utilized to assess the parents’ acceptance (e.g., “My father/mother talk to me in a warm and affectionate way”) and 16 items in hostility/aggression, indifference/neglect, and undifferentiated rejection subscales were used to assess the parents’ rejection (e.g., “My father/mother nag or scold me when I am bad”). Global scores of parents’ acceptance and rejection were computed for each of the parents, with higher scores indicating greater parents’ acceptance or rejection of the child. Participants rated how often their own parents used each discipline strategy during the year that they were before 16-year-old.

The Chinese version of PARQ was used widely and showed good internal reliability and validity (Putnick et al., 2015; Xing et al., 2019). In the present study, the Cronbach’s alpha coefficients for mothers’ and fathers’ reports of acceptance were 0.86 and 0.86, respectively. The Cronbach’s alpha coefficients for mothers’ and fathers’ reports of rejection were 0.88 and 0.89, respectively.

#### Undergraduate adjustment to college

The College Student Adaptability Inventory (CSAI) was used to assess the undergraduate adjustment to college (Lu, 2003). Sixty items are grouped into seven subscales: learning adaptability measures students’ ability to cope with the educational demands of college (8 items, e.g., I find it hard to get used to the learning style at the university), interpersonal adaptability refers to students’ ability to build harmonious relationships (11 items, e.g., I have the ability to handle relationships with classmates independently), social role adaptability measures students’ ability to adjust their mind and behaviors independently to meet the requirements of the new role (9 items, e.g., I have the ability to adapt to changing group status), career choice adaptability represents students’ ability to manage and plan their future career choice, which is also referred to as career preparation (9 items, e.g., I have a clear career interest), livelihood self-management adaptability refers to students’ ability to organize their daily life in university without being under the care of their parents (6 items, e.g., I have the ability to manage my money and plan my budget in university), environmental general adaptability quantifies a general sense of how a student feels physically and psychologically (7 items, e.g., I think the hardware facilities in our university are very poor), and somatic-mental symptom measures students’ adverse physical and psychological symptoms when completing the above adaptation tasks (10 items, e.g., I’m always out of spirits). In addition, the CSAI also provides six repeat items to verify the validity of each questionnaire. Participants rated each item on a 5-point Likert scale, responding on a range from 1 = strongly disagree to 5 = strongly agree. A higher score represents a higher level of undergraduate adjustment to college.

The Chinese version of the CSAI has been widely used and has shown good internal reliability and validity (Niu et al., 2022). In the present study, the Cronbach’s *α* for the seven subscales ranged from 0.81 to 0.89.

#### Undergraduate collectivism

The Individualism–Collectivism Scale (ICS) was used to assess the undergraduate collectivism (Triandis and Gelfand, 1998; Triandis, 2001), which is a 16-item scale designed to measure four dimensions of collectivism and individualism: horizontal collectivism (e.g., It is a pleasure for me to spend time with other people), vertical collectivism (e.g., It is important to me to respect the collective decision), horizontal individualism (e.g., I rely on myself rather than others in most of the time), and vertical individualism (e.g., It is important to me to be better than everyone else). The participants rated the extent to which they agreed with the statements in the survey (1 = strongly disagree, 9 = strongly agree). In the present study, the horizontal collectivism subscale and vertical collectivism subscale scores were summed to form a total score for undergraduate collectivism.

The Chinese version of the ICS has been widely used and has shown good internal reliability and validity (Chiou, 2010). In the present study, the Cronbach’s α for the undergraduate collectivism was 0.81.

### Control of common method variance

Considering that parents’ acceptance, parents’ rejection, undergraduate adjustment to college, and undergraduate collectivism were all reported by undergraduates, A series of procedural remedies were conducted to minimize the effects of common method variance, such as adopting mature scales and setting a time lag between the measurements (Podsakoff et al., 2003). In addition, Harman’s single-factor test, a widely used method, were employed to detect the threat of common method variance (Podsakoff and Organ, 1986). Factor analysis were performed on all items, and found that 27 factors with eigenvalues greater than one were extracted from the data, with the first factor accounting for 22.67% of the variance (less than 40%). These results suggested that common method variance did not appear to be a problem in this study.

### Data analysis

The SPSS 21.0 for Windows was used for data analyses. Prior to conducting analyses, normality of data distribution was examined. Analysis of skewness and kurtosis indicated that parents’ acceptance, parents’ rejection, undergraduate adjustment to college, and undergraduate collectivism were all normal distribution (−0.93 < *skew* < 1.56; −0.57 < *kurtosis* < 2.96). In the present study, all analyses were performed separately for parents’ acceptance and parents’ rejection. Data analyses proceeded in two stages. First, bivariate correlation analyses were used to explore the relations between parents’ parenting and undergraduate adjustment to college. Second, a series of hierarchical regression analyses were performed to investigate whether the relations between parents’ parenting and undergraduate adjustment to college moderated by undergraduate collectivism. Child age, child gender and family SES were included as covariates in the first step. The fathers’ acceptance (or rejection), and mothers’ acceptance (or rejection) and undergraduate collectivism were entered simultaneously in the second step. The interactions between fathers’ acceptance (or rejection) × undergraduate collectivism and mothers’ acceptance (or rejection) × undergraduate collectivism were entered in the third step. When significant interactions were found, the nature of the interaction was tested by follow-up simple slopes analyses, conducted as recommended by Holmbeck (2002). Following Aiken and West (1991), prior to conducting the analyses, all of the predictors were mean centered to reduce multicollinearity.

## Results

### Preliminary analyses

The descriptive statistics for all the measures included in the present study and the results of the bivariate correlation analyses were presented in Table 1. As shown in Table 1, For both mothers and fathers, the bivariate correlation analyses indicated that higher levels of parents’ acceptance were linked to higher levels of undergraduate adjustment to college, whereas higher levels of parents’ rejection were linked to lower levels of undergraduate adjustment to college. Moreover, the bivariate correlation analyses also indicated that a higher level of undergraduate collectivism was related with a higher level of undergraduate adjustment to college.

**Table 1:** The mean (*M*), standard deviation (*SD*), and the correlations among the variables.

	1	2	3	4	5	6	7	8	9
1 Child gender (1 = boy, 0 = girl)									
2 Child age	0.03^*^								
3 SES	−0.01	−0.09^**^							
4 Fathers’ acceptance	0.00	0.01	−0.04^**^						
5 Mothers’ acceptance	−0.00	0.01	−0.05^***^	0.85^***^					
6 Fathers’ rejection	0.02	−0.02	0.03	−0.65^***^	−0.58^***^				
7 Mothers’ rejection	0.03^*^	−0.01	0.03	−0.57^***^	−0.63^***^	0.92^***^			
8 Undergraduate collectivism	0.03^*^	−0.00	−0.02	0.35^***^	0.37^***^	−0.27^***^	−0.26^***^		
9 Undergraduate adjustment to college	0.2	0.00	−0.03^*^	0.50^***^	0.50^***^	−0.48^***^	−0.49^***^	0.36^***^	
*M*		18.15	11.39	26.01	27.05	22.92	22.19	51.57	233.40
*SD*		0.54	3.91	4.67	4.24	6.75	6.23	11.96	33.21

^*^p < 0.05; ^**^p < 0.01; ^***^p < 0.001. Two-tailed tests.

### Hierarchical regression analyses

A series of hierarchical regression analyses were conducted separately for parents’ acceptance and parents’ rejection to evaluate the moderating effects of undergraduate collectivism on the relations between parents’ parenting and undergraduate adjustment to college. As shown in Table 2, similar results of the regression model were found for parents’ acceptance and parents’ rejection in the present study. On the one hand, for parents’ acceptance, the regression analyses revealed that higher levels of fathers’ acceptance, mothers’ acceptance, and undergraduate collectivism significantly predicted higher levels of undergraduate adjustment to college. Moreover, undergraduate collectivism significantly moderated the association between fathers’ acceptance and undergraduate adjustment to college, but it did not moderate the association between mothers’ acceptance and undergraduate adjustment to college. On the other hand, for parents’ rejection, the regression analyses revealed that lower levels of fathers’ rejection, mothers’ rejection, and higher levels of undergraduate collectivism significantly predicted higher levels of undergraduate adjustment to college. Moreover, undergraduate collectivism significantly moderated the association between fathers’ rejection and undergraduate adjustment to college, but it did not moderate the association between mothers’ rejection and undergraduate adjustment to college. Further, the follow-up simple slopes analyses were conducted to test the nature of the interactions. In the present study, values at 1 *SD* above and below the mean of undergraduate collectivism were used to calculate the simple slopes of the association between fathers’ parenting and undergraduate adjustment to college.

**Table 2:** Regression analyses testing the moderating effects of undergraduate collectivism on the relations between parents’ acceptance/ rejection and undergraduate adjustment to college.

Criterion variable	Predictor	β	*t*	95% CI	*ΔR* ^2^	*F*
Parents’ acceptance						
Step 1	Gender (1 = boy, 0 = girl)	0.02	1.26	[−0.66 3.03]	0.00	2.39
	Age	0.01	0.65	[−1.10 2.19]		
	SES	−0.03	−2.22^*^	[−0.48–0.03]		
Step 2	Fathers’ acceptance	0.25	11.28^***^	[1.45 2.06]	0.30	392.51^***^
	Mothers’ acceptance	0.21	9.73^***^	[1.34 2.02]		
	Undergraduate collectivism	0.20	16.18^***^	[0.48 0.62]		
Step 3	Fathers’ acceptance × Undergraduate collectivism	0.08	3.10^**^	[0.02 0.07]	0.01	306.19^***^
	Mothers’ acceptance × Undergraduate collectivism	0.02	0.88	[−0.02 0.04]		
Parents’ rejection						
Step 1	Gender (1 = boy, 0 = girl)	0.02	1.26	[−0.66 3.03]	0.00	2.39
	Age	0.01	0.65	[−1.10 2.19]		
	SES	−0.03	−2.22^*^	[−0.48–0.03]		
Step 2	Fathers’ rejection	−0.22	−7.77^***^	[−1.38–0.82]	0.30	388.90^***^
	Mothers’ rejection	−0.21	−7.36^***^	[−1.43–0.83]		
	Undergraduate collectivism	0.24	20.41^***^	[0.61 0.74]		
Step 3	Fathers’ rejection× Undergraduate collectivism	−0.08	−2.38^*^	[−0.05–0.01]	0.01	301.35^***^
	Mothers’ rejection× Undergraduate collectivism	−0.01	−0.43	[−0.03 0.02]		

^*^p < 0.05; ^**^p < 0.01; ^***^p < 0.001. Two-tailed tests.

**Figure 1:**
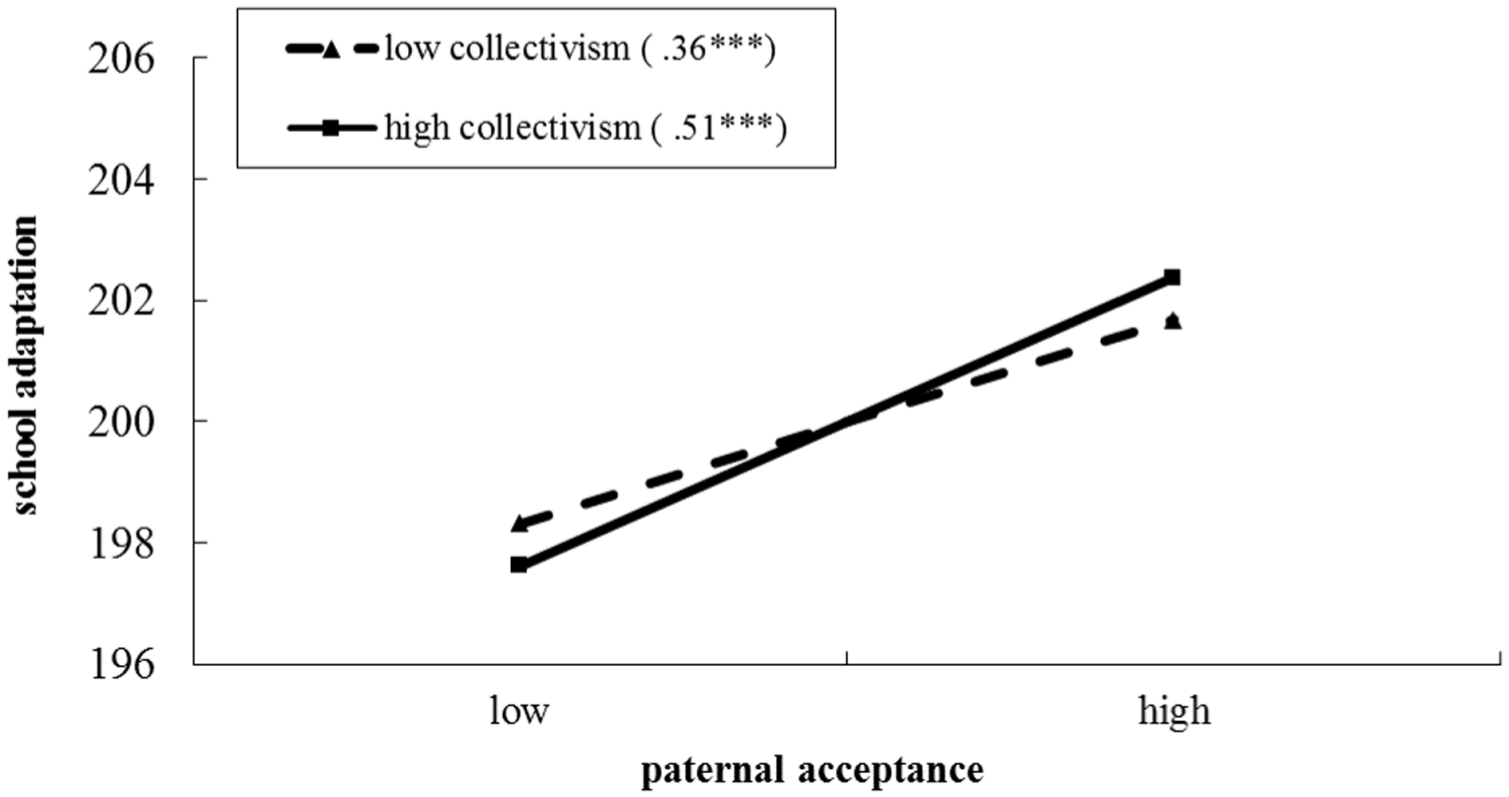
The moderating effects of undergraduate collectivism on the association between fathers’ acceptance and undergraduate adjustment to college. The numbers in parentheses are simple slopes. ^***^p < 0.001. Two-tailed tests.

Regarding to the moderating effects of undergraduate collectivism on the association between fathers’ acceptance and undergraduate adjustment to college, the hierarchical regression model was significant, explaining 31.1% of the variance in undergraduate adjustment to college. The results of simple slopes analyses emerged a pattern consistent with an intensifying process. As illustrated in Figure 1, the fathers’ acceptance was more strongly associated with undergraduate adjustment to college when undergraduate collectivism was high (*β* = 0.51, *t* = 30.47, *p* < 0.001), than when undergraduate collectivism was low (*β* = 0.36, *t* = 24.08, *p* < 0.001). That is, it was found that high undergraduate collectivism may act as a positive factor for the impact of fathers’ acceptance on undergraduate adjustment to college.

**Figure 2:**
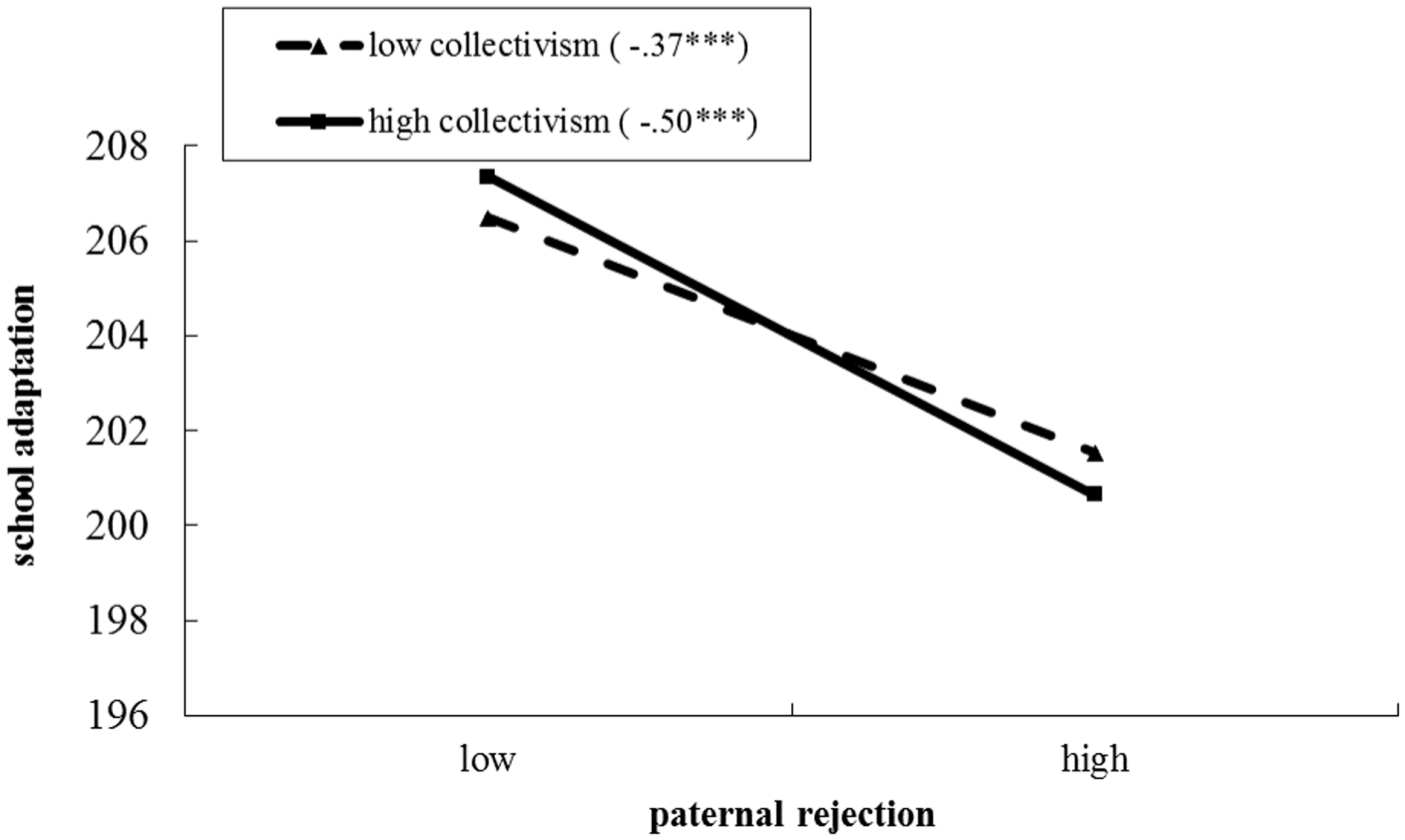
The moderating effects of undergraduate collectivism on the association between fathers’ rejection and undergraduate adjustment to college. The numbers in parentheses are simple slopes. ^***^p < 0.001. Two-tailed tests.

Regarding to the moderating effects of undergraduate collectivism on the association between fathers’ rejection and undergraduate adjustment to college, the hierarchical regression model was significant, explaining 30.7% of the variance in undergraduate adjustment to college. The results of simple slopes analyses emerged a pattern consistent with a buffering process. As illustrated in Figure 2, the fathers’ rejection was more strongly associated with undergraduate adjustment to college when undergraduate collectivism was high (*β* = −0.50, *t* = −28.87, *p* < 0.001), than when undergraduate collectivism was low (*β* = −0.37, *t* = −26.48, *p* < 0.001). That is, it was found that high undergraduate collectivism may act as a risk factor for the impact of fathers’ rejection on undergraduate adjustment to college.

## Discussion

The present study expanded on previous research which investigate the impact of cultural values on the relation between parenting and individual development by examining the moderating effects of undergraduate’s collectivism on the relations between both mothers’ and fathers’ parenting (acceptance and rejection) and undergraduate adjustment to college with a relatively large sample of Chinese undergraduate (Markus and Kitayama, 1991; Trentacosta and Shaw, 2009; Peters et al., 2011; Putnick et al., 2015; Huppert et al., 2018; Ramírez-Uclés et al., 2018). To the best of our knowledge, this study is the first large-scale survey to explore these issues. For hypothesis 1, the findings from this study indicated that both fathers’ and mothers’ acceptance positively influenced the undergraduate adjustment to college, while both fathers’ and mothers’ rejection had a negative impact on the undergraduate adjustment to college. Moreover, for hypothesis 2 and 3, regarding to the results of the moderating role of undergraduate’s collectivism, somewhat different patterns of fathers’ model and mothers’ model emerged. This study found undergraduate’s collectivism moderated the relations between parenting (acceptance and rejection) and undergraduate adjustment to college for fathers, but not mothers. These aspects of the results will be discussed below.

The present study extended past research by examining the relations between parents’ parenting (acceptance and rejection) and undergraduate adjustment to college with a relatively large sample of undergraduate. Consistent with previous research (Khaleque and Rohner, 2002, 2004; Hale et al., 2008; Dwairy, 2010), the present study found that both fathers’ and mothers’ acceptance in childhood positively influenced the undergraduate adjustment to college, while both fathers’ and mothers’ rejection in childhood had a negative impact on the undergraduate adjustment to college. Combined the results of this study and previous research, such results demonstrated that parents’ acceptance and rejection not only have immediate effects, but also have long-term effects on individuals’ adjustment. The reason may be that, on the one hand, parents’ acceptance may enhance, whereas parents’ rejection can reduce the children’s mental resilience (Ogelman, 2015; Sart et al., 2016). On the other hand, parental rejection may be also enhance the children’s sensitivity to rejection to a certain extent (Rowe et al., 2014). Within this context, undergraduate who experienced parents’ rejection may be sensitive to interpersonal rejection and poor in mental resilience, which in turn may lead them more susceptible to challenge or risk, and more difficult in adjust themselves’ mental health. Thus, when they must adjust to a new school environment, build new interpersonal relationships, leave the nest, and assume personal responsibility for the first time in the university (Larose et al., 2019), such challenges may easily cause social, emotional, and learning maladjustment (Leary and DeRosier, 2012; Rogers et al., 2018), resulting in difficulties in school adaptation. On the contrary, since undergraduate who experienced parents’ acceptance are insensitive to interpersonal rejection and good in mental resilience, they may be more ability to overcome challenges and good at adjust themselves’ mental health, resulting in high levels of adjustment to college.

The present study also extended past research by examining the moderating effect of undergraduate’s collectivism on the relations between both mothers’ and fathers’ parenting (acceptance and rejection) and undergraduate adjustment to college with a relatively large sample of Chinese undergraduate. Partially consistent with the initial expectations, the present research found that undergraduate’s collectivism moderated the relations between acceptance/rejection and undergraduate adjustment to college for fathers, but not mothers. To be specific, compared to undergraduate low in collectivism, those high in collectivism experienced a stronger positive impact from fathers’ acceptance and a more pronounced negative effect from fathers’ rejection. These results were inconsistent with the existing cross-cultural research and research about familism value mentioned above. Possibly, regarding to the cross-cultural research, to date most existing cross-cultural research conceptualized that individuals in the same cultural group hold the same cultural values, and examined the impact of collectivism by examining the differences between cultural group. In fact, the differences between cultural groups extend beyond collectivism, as various factors may collaborate to create a protective effect on adjustment. Analogously, familism value also include many other factors related to adjustment besides collectivism including compliance and obedience to parents, value family as a source of attachment and support, and loyalty and obligation (Cahill et al., 2021), which together create a protective effect on adjustment.

Therefore, to some extent, these results provided further evidence that “pure” collectivism acted as an amplifier factor rather than a protective factor, in the relations between parenting and child development (Markus and Kitayama, 1991; Oyserman et al., 2002; Huppert et al., 2018). Further, the pattern of moderating effect of collectivism from this study is quite similar to the differential susceptibility model (Belsky and Pluess, 2009). The differential susceptibility model highlights the importance of individual variations in developmental plasticity. It suggests that individuals with high plasticity are more influenced by both positive and negative aspects of their social environments, while those with low plasticity exhibit less biobehavioral responsiveness to their surroundings regardless of the nature of the stimuli (Belsky and Pluess, 2009). Thus, from the perspective of the differential susceptibility model, collectivism, like other individual factors found in previous studies, makes individuals more plastic in their growth and thus more susceptible to the influence of environmental factors. This result suggests that environmental factors such as parenting have more significant impact on collectivists than on individualists. Therefore, it is particularly important to provide collectivists’ parents with more positive and effective family education guidance.

It is noteworthy that the pattern of moderating effects of collectivism were differ by parent gender, undergraduate’s collectivism moderated the relations between parenting (acceptance and rejection) and undergraduate adjustment to college for fathers, but not mothers. This can be interpreted according to the role that Chinese parents play in child rearing. Typically, mothers are still regarded as important for providing care and affection to the child in contemporary Chinese families (Ho, 1987; Wong et al., 2009), although Chinese fathers have become more actively engaged in their children’s lives in recent years (Chuang and Su, 2008; Xing and Wang, 2018). Just as the study by Ma et al. (2012) indicated that modernization and westernization have changed parental roles into “an under-involved father and an over-involved mother.” In this case, compared with fathers, mothers spend more time in caregiving children and more often act as the primary caregivers and therefore their acceptance or rejection has more stable and stronger relation with child development, which may be less moderated by other factors such as collectivism. Conversely, since fathers spend less time caring for their children, their disciplinary behavior has less opportunity to have an impact on individual development, making the effects of discipline less stable. In this case, compared with individuals low in collectivism, individuals high in collectivism are more likely to recognize and be affected by their father’s acceptance or rejection due to their high dependence and sensitivity to the environment. As a result, undergraduate’s collectivism moderated the relations between parenting (acceptance and rejection) and undergraduate adjustment to college for fathers, but not mothers.

Several limitations of this study should be noted. First, the present study used single informants for information about parents’ acceptance and rejection, undergraduate adjustment to college and undergraduate’s collectivism. Despite procedural remedies were adopted to minimize the effects of common method variance and Harman’s single-factor tests suggested that common method variance did not appear to be a problem in the present study, utilizing the reports of as many reliable informants as possible to increase the reliability of the measurement would be useful in the future research. Second, due to the cross-sectional approach, the present study may be subject to retrospective inaccuracies. For example, undergraduates may only be able to recall some parenting situations that are meaningful or memorable to them, leading to underestimations of parents’ acceptance/rejection levels. Therefore, a longitudinal study examining the actual relations between parenting (acceptance and rejection) and undergraduate adjustment to college should be conducted in the future. Third, a relatively homogenous sample which primarily consisted of Chinese middle-class families with undergraduate was used the present study. Hence, future studies should examine to what extent the present results can be generalized to other families from different social and cultural backgrounds.

Despite these limitations, some important practical implications and valuable information can be derived from the present study. First, to the best of our knowledge, this study is the first large-scale survey to explore the moderating effects of undergraduate’s collectivism on the relations between both mothers’ and fathers’ parenting (acceptance and rejection) and undergraduate adjustment to college. Given the dearth of research examining the mechanisms underlying the parenting processes, understanding the mechanisms of these processes is extremely valuable for improving the intervention of family education guidance. Second, the present study demonstrated that both fathers’ and mothers’ acceptance positively influenced the undergraduate adjustment to college, while both fathers’ and mothers’ rejection had a negative impact on the undergraduate adjustment to college. Combined these results and previous research, such results demonstrated that parents’ acceptance and rejection not only have immediate effects, but also have long-term effects on individuals’ adjustment. Prevention programs designed to identify and provide services for undergraduate based on their experiences of parents’ rejection aggression and could potentially improve their adjustment to college.

Third, the present study demonstrated that compared to undergraduate low in collectivism, those high in collectivism experienced a stronger positive impact from fathers’ acceptance and a more pronounced negative effect from fathers’ rejection. This result suggests that environmental factors such as parenting have more significant impact on collectivists than on individualists. Therefore, it is particularly important to provide collectivists’ parents with more positive and effective family education guidance. Final, although the pattern of moderating effects of collectivism were differ by parent gender, the direct effects of acceptance and rejection of fathers and mothers on undergraduate adjustment to college are stable. Therefore, providing practical and effective family education guidance and helping parents adopt more scientific, effective, and highly acceptance parenting methods is the most effective way to improve the school adjustment of future generations.

## Data Availability

The raw data supporting the conclusions of this article will be made available by the authors, without undue reservation.
